# Unravelling the Molecular Identity of Bulgarian Jumping Plant Lice of the Family Aphalaridae (Hemiptera: Psylloidea)

**DOI:** 10.3390/insects15090683

**Published:** 2024-09-10

**Authors:** Monika Pramatarova, Daniel Burckhardt, Igor Malenovský, Ilia Gjonov, Hannes Schuler, Liliya Štarhová Serbina

**Affiliations:** 1 Department of Zoology and Anthropology, Faculty of Biology, Sofia University, Dragan Tzankov 8, 1164 Sofia, Bulgaria; gjonov@cicadina.com; 2 Naturhistorisches Museum, Augustinergasse 2, 4001 Basel, Switzerland; daniel.burckhardt@bs.ch; 3 Department of Botany and Zoology, Faculty of Science, Masaryk University, Kotlářská 2, 611 37 Brno, Czech Republic; malenovsky@sci.muni.cz; 4 Faculty of Agricultural, Environmental and Food Sciences, Free University of Bozen-Bolzano, 39100 Bolzano, Italy; hannes.schuler@unibz.it (H.S.); liliia.serbina@mfn.berlin (L.Š.S.); 5 Competence Center for Plant Health, Free University of Bozen-Bolzano, 39100 Bolzano, Italy; 6 Centre for Integrative Biodiversity Discovery, Museum für Naturkunde, 10115 Berlin, Germany

**Keywords:** distance-based method, DNA barcoding, phylogeny, psyllids, rapid species identification, sequence database

## Abstract

**Simple Summary:**

Correct taxonomic identification is essential for conducting successful biological research, especially with regard to economically important insects, such as jumping plant lice or psyllids. In the present study, we identify and diagnose the morphologically characterised aphalarid species from Bulgaria using two molecular markers, cytochrome c oxidase I and cytochrome b. A total of 80 sequences of 25 Aphalaridae species were obtained and included in the BOLD and GenBank databases. This should enable even non-experts to identify these species quickly and accurately. The results of the current study show that two barcode genes are sufficient to distinguish most aphalarid species.

**Abstract:**

Psyllids (Hemiptera: Psylloidea) are plant sap-sucking insects whose identification is often difficult for non-experts. Despite the rapid development of DNA barcoding techniques and their widespread use, only a limited number of sequences of psyllids are available in the public databases, and those that are available are often misidentified. Here, we provide 80 sequences of two mitochondrial genes, cytochrome c oxidase I (*COI*) and cytochrome b (*Cytb*), for 25 species of Aphalaridae, mainly from Bulgaria. The DNA barcodes for 15 of these species are published for the first time. In cases where standard primers failed to amplify the target gene fragment, we designed new primers that can be used in future studies. The distance-based thresholds for the analysed species were between 0.0015 and 0.3415 for *COI* and 0.0771 and 0.4721 for *Cytb*, indicating that the *Cytb* gene has a higher interspecific divergence, compared to *COI*, and therefore allows for more accurate species identification. The species delimitation based on DNA barcodes is largely consistent with the differences resulting from morphological and host plant data, demonstrating that the use of DNA barcodes is suitable for successful identification of most aphalarid species studied. The phylogenetic reconstruction based on maximum likelihood and Bayesian inference analyses, while showing similar results at high taxonomic levels to previously published phylogenies, provides additional information on the placement of aphalarids at the species level. The following five species represent new records for Bulgaria: *Agonoscena targionii*, *Aphalara affinis*, *Colposcenia aliena*, *Co. bidentata*, and *Craspedolepta malachitica*. *Craspedolepta conspersa* is reported for the first time from the Czech Republic, while *Agonoscena cisti* is reported for the first time from Albania.

## 1. Introduction

The accurate identification of species is key to successful biological research. Over the last three decades, molecular methods have become increasingly popular, often replacing taxonomic expertise founded in morphology [1]. DNA barcoding is expected to provide a rapid and reliable method for species identification, especially for specimens lacking conspicuous morphological features (e.g., immature insects, cryptic species) or species exhibiting seasonal or sexual dimorphism [2,3]. Consequently, molecular identification is now widely used for the detection of quarantine species and plant pests [4,5,6]. However, the use of DNA barcoding for reliable species identification is only possible if the gene sequences of correctly identified individuals are available in reference databases such as BOLD or GenBank [7]. Unfortunately, these databases are quite incomplete for many groups and are plagued by misidentifications [8] as they lack a quality control mechanism.

Jumping plant lice or psyllids (Hemiptera: Psylloidea) constitute a group of Sternorrhyncha. Some psyllid species are serious pests of crops or ornamental plants, while others are used as biological control agents [9,10,11,12]. Psyllids are small (1–10 mm body length, including the wings when folded over the body), sap-sucking, generally host-specific insects that are predominantly associated with eudicots and magnoliids and only exceptionally with monocots or conifers [13,14]. Slightly more than 4000 species have been described from all biogeographical regions, except Antarctica, but at least as many are still undescribed [14]. From Europe, a comparatively well-studied region, about 400 species are known, representing six (Aphalaridae, Calophyidae, Carsidaridae, Liviidae, Psyllidae, and Triozidae) of the seven currently recognised psyllid families [15]. The molecular data available in GenBank “https://www.ncbi.nlm.nih.gov/genbank/ (accessed on 12 November 2023)” and BOLD “http://boldsystems.org (accessed on 12 November 2023)” mostly concern pest psyllids such as *Bactericera cockerelli* (Šulc), pear psyllids of the genus *Cacopsylla* Ossiannilsson, and *Diaphorina citri* Kuwayama, while information on non-pest psyllids is often limited [6,16].

Aphalaridae are the third largest family of Psylloidea, with 770 described species [15]. Their phylogeny is still controversial as to whether they are monophyletic or paraphyletic [16,17]. Wang et al. [18], however, provided evidence in favour of the monophyly of the family. Burckhardt et al. [14] recognised seven subfamilies worldwide, of which only Aphalarinae and Rhinocolinae are native to Europe and Bulgaria, while several introduced pests of *Eucalyptus* sp. from Spondyliaspidinae also occur in Europe [19,20,21,22]. The monophyly of Aphalarinae and Rhinocolinae is strongly supported by molecular and morphological data ([14] and the literature cited therein). The phylogenetic relationships within the two subfamilies have been analysed at the generic level using morphological data [23,24,25] and for a limited set of taxa using molecular data [16,17].

In Bulgaria, Aphalaridae are represented by 20 species of Aphalarinae [26,27,28,29,30,31,32,33,34,35,36] and four species of Rhinocolinae [23,28]. *Aphalara* Foerster and *Craspedolepta* Enderlein are two species-rich genera of Aphalarinae with a predominantly Holarctic distribution. In the past, both genera were divided into two or more genera or subgenera, which led to artificial groupings, as shown by Burckhardt and Lauterer [32], who defined the genera as putatively monophyletic.

Due to minor morphological differences and overlapping host ranges between some species, the identification of Aphalaridae, especially *Aphalara*, *Craspedolepta*, and *Agonoscena* Enderlein, may prove difficult for non-experts. Here, we evaluate the accuracy and efficiency of DNA barcoding of the two protein-coding mitochondrial gene fragments *COI* and *Cytb* for the identification of 25 species of Aphalaridae (23 spp. from Bulgaria, 1 sp. from the Czech Republic, 1 sp. from Albania). We also use the newly obtained molecular data to shed more light on the phylogenetic relationships within Aphalaridae.

## 2. Materials and Methods

### 2.1. Material

**Figure 1:**
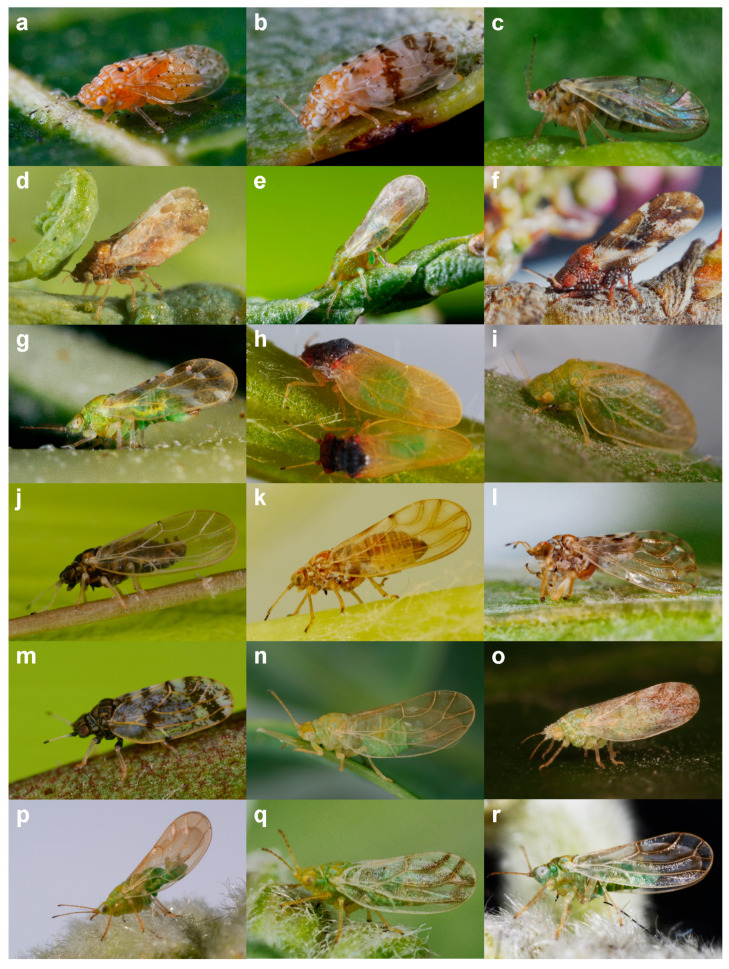
Habitus of the studied species of Aphalaridae from Bulgaria. (**a**) *Agonoscena targionii*; (**b**) *A. pistaciae*; (**c**) *Megagonoscena gallicola*; (**d**) *Colposcenia aliena*; (**e**) *Co. bidentata*; (**f**) *Co. osmanica*; (**g**) *Co. traciana*; (**h**) *Rhodochlanis bicolor*; (**i**) *Rhinocola aceris*; (**j**) *Aphalara affinis*; (**k**) *Aph. avicularis*; (**l**) *Aph. maculipennis*; (**m**) *Aph. nigrimaculosa*; (**n**) *Craspedolepta bulgarica*; (**o**) *Cr. conspersa*, specimen from Austria, photo credit to T. Oswald; (**p**) *Cr. innoxia*; (**q**) *Cr. nervosa*; (**r**) *Cr. pontica*.

A total of 75 specimens of 25 Aphalaridae species were collected from 27 localities in Bulgaria (23 spp.), Albania (1 sp.), and the Czech Republic (1 sp.) (Table 1 and Figure 1). The specimens were collected during faunistic surveys in different types of vegetation using a sweep net and an aspirator. The host plants were identified in the field and are listed in Table 1. Specimens collected after 2016 were preserved in 70% or 96% ethanol, while specimens collected before 2016 were mounted dry. The material was primarily identified on the basis of morphological characters of the adults according to the identification keys and taxonomic descriptions of Ossiannilsson [37] and Burckhardt and Lauterer [32] for *Aphalara* spp., Loginova [38] and Burckhardt [39] for *Colposcenia* Enderlein, Ossiannilsson [37], Loginova [40,41], Dobreanu and Manolache [42], Conci and Tamanini [43], and Burckhardt and Lauterer [44] for *Craspedolepta*, Conci and Tamanini [45] and Burckhardt [46] for *Rhodochlanis* Loginova, and Burckhardt and Lauterer [23] for Rhinocolinae. The nomenclature and classification follow the Psyl’list database [15]. The voucher specimens are deposited in the Zoological Collection of the University of Sofia.

### 2.2. DNA Extraction, Amplification, Sequencing and Alignment

DNA extraction, amplification, and sequencing of the *COI* gene fragment of 21 species were performed by the Canadian Centre for DNA Barcoding (CCDB) using non-destructive DNA extraction from the whole specimen. The non-destructive method was chosen to preserve the morphological integrity of these specimens for future analysis. Later, nineteen samples of 18 species were destructively extracted with DNeasy Blood and Tissue (QIAGEN) in the molecular laboratory of the Free University of Bozen-Bolzano according to the manufacturer’s protocol. The fragments of the two mitochondrial genes *COI* and *Cytb* were amplified there with DreamTaq DNA Polymerase (Thermo Fisher, Waltham, MA, USA). We chose these two genes because of their utility in species identification and phylogeographic studies, with *COI* being widely used for DNA barcoding and *Cytb* often used as an additional marker [2,4,8,47,48]. We designed new primers and developed protocols for the amplification of *Cytb* when the universal primer pairs *Cytbf*/*Cytbr* [16,49] failed to amplify the targeted gene fragment. The newly developed primers were designed manually using the conserved gene regions. Their suitability and the compatibility of the forward and reverse primer pairs were assessed using the PCR Primer Stats tools “https://www.bioinformatics.org/sms2/pcr_primer_stats.html (accessed on 15 December 2023)”. All primer sequences and PCR conditions are listed in Table 2. PCR products were purified and Sanger sequenced by Eurofins Genomics (Ebersberg, Germany). For the following seven species, *Agonoscena pistaciae*, *Aphalara freji*, *Aph. polygoni*, *Craspedolepta bulgarica*, *Cr. conspersa*, *Cr. innoxia*, and *Cr. omissa*, only the *COI* gene fragment was sequenced as no additional DNA was available.

The PCR products were sequenced in both directions. The sequences were aligned and trimmed using Geneious v.8.1.9 (Dotmatics, Boston, MA, USA) [50]. The alignment of the sequences was performed manually with MEGA v.11 [51]. The gene sequences were concatenated with SEAVIEW v.5.0.5 [52].

**Table 1:** Collecting data for the specimens of Aphalaridae associated with DNA barcodes or included in the phylogenetic analyses, with references to BOLD and GenBank databases. Abbreviations used in the table: M: male, F: female. The accession numbers of the sequences that were sequenced for the first time, are printed in bold. Psyllid species that represent new records for Bulgaria, Albania, or the Czech Republic are marked with an asterisk (*). For some voucher entries, only limited information about the locality is available in GenBank.

Subfamily	Species	Locality	Host Plant	Latitude	Longitude	Altitude (m)	Collection Date	Sex	Sofia University Catalog Number	Process ID (BOLD)	Barcode Index Number (BIN)	Accession N (GenBank)	Reference	Figure
Aphalarinae	* *Aphalara affinis* (Zetterstedt, 1828)	Bulgaria, Western Rhodopi Mts., Smolyanski ezera lakes	-	41.6203	24.6771	1520	15 September 2021	M	BFUS-I-IG026770	PSYBG001-23	BOLD:AFM2800	**PQ109732**	This study	Figure 1j
		Bulgaria, Western Rhodopi Mts., Smolyanski ezera lakes	-	41.6203	24.6771	1520	15 September 2021	M	BFUS-I-IG026975	PSYBG100-24	-	**PQ100053**	This study	Figure 1j
Aphalarinae	*Aphalara itadori* (Shinji, 1938)	Japan	-	-	-	-	-	-	-	-	-	KP113670	[53]	-
		United Kingdom	-	-	-	-	-	-	-	-	-	MG988914	[16]; Percy, pers. comm.	-
Aphalarinae	*Aphalara avicularis* Ossiannilsson, 1981	Bulgaria, Western Stara Planina Mts., Churek	*Polygonum aviculare* L.	42.7767	23.7157	796	27 August 2023	M	BFUS-I-IG026773	PSYBG004-23	BOLD:ACY0265	**PQ109737**	This study	Figure 1k
		Bulgaria, Western Stara Planina Mts., Churek		42.7767	23.7157	796	27 August 2023	F	BFUS-I-IG026977	PSYBG115-24	-	**PQ100054**	This study	Figure 1k
Aphalarinae	*Aphalara freji* Burckhardt & Lauterer, 1997	Bulgaria, Eastern Stara Planina Mts., Bardarevo	-	42.8983	27.6219	72	27 July 2011	M	BFUS-I-IG005891	PSYBG062-23	BOLD:ACY0265	**PQ109739**	This study	-
Aphalarinae	*Aphalara maculipennis* Löw, 1886	Bulgaria, Western Stara Planina Mts., Aldomivsko lake	-	42.8848	22.9998	660	13 May 2022	F	BFUS-I-IG026960	PSYBG096-24	BOLD:AFT1810	**PQ109740** **PQ100055**	This study	Figure 1l
Aphalarinae	*Aphalara nigrimaculosa* Gegechkori, 1981	Bulgaria, Western Rhodopi Mts., Pamporovo, Snezhanka peak	*Rumex acetosella* L.	41.6370	24.6835	1850	16 September 2021	M	BFUS-I-IG026982	PSYBG097-24	BOLD:AFT1810	**PQ109742** **PQ100056**	This study	Figure 1m
Aphalarinae	*Aphalara polygoni* Foerster, 1848	Bulgaria, Western Rhodopi Mts., Cigov chark	-	41.9318	24.1292	1380	18 June 2016	F	BFUS-I-IG005903	PSYBG063-23	BOLD:AFL6229	**PQ109743**	This study	-
Aphalarinae	* *Colposcenia aliena* (Löw, 1881)	Bulgaria, East Danube plain, Poveljanovo district	*Tamarix* sp.	43.2122	27.6480	74	6 July 2023	M	BFUS-I-IG026791	PSYBG016-23	BOLD:AFN1581	**PQ109745**	This study	Figure 1d
		Bulgaria, East Danube plain, Poveljanovo district		43.2122	27.6480	74	6 July 2023	M	BFUS-I-IG027010	PSYBG102-24	-	**PQ100058**	This study	Figure 1d
Aphalarinae	* *Colposcenia bidentata* Burckhardt, 1988	Bulgaria, Eastern Rhodopi Mts., Meden buk	*Tamarix* sp.	41.3696	26.0545	110	6 July 2012		BFUS-I-IG020788	PSYBG066-23	BOLD:AFN1583	**PQ109747**	This study	-
		Bulgaria, Eastern Rila-Rhodopi Massif, near Arda river		41.6514	25.8687	140	27 August 2022	F	BFUS-I-IG027009	PSYBG103-24	-	**PQ100059**	This study	Figure 1e
Aphalarinae	*Colposcenia osmanica* Vondráček, 1953	Bulgaria, Vlahina Planina Mts., Simitli	*Tamarix* sp.	41.8945	23.1184	290	8 May 2022	M	BFUS-I-IG026785	PSYBG019-23	BOLD:AFN1582	**PQ109749**	This study	-
		Bulgaria, Krumovgrad		41.5044	25.3948	255	10 October 2023	M	BFUS-I-IG027006	PSYBG104-24	-	**PQ100060**	This study	-
Aphalarinae	*Colposcenia traciana* (Klimaszewski, 1970)	Bulgaria, Strandzha Mts., Tsarevo	*Tamarix* sp.	42.1363	27.7992	142	12 July 2023	M	BFUS-I-IG026788	PSYBG022-23	BOLD:AFL7588	**PQ109752**	This study	Figure 1g
		Bulgaria, Strandzha Mts., Tsarevo		42.1363	27.7992	142	12 July 2023	M	BFUS-I-IG027003	PSYBG105-24	-	**PQ100061**	This study	Figure 1g
Aphalarinae	*Colposcenia* sp.	Bulgaria, Meden buk	-	41.3696	26.0545	110	6 July 2012	-	-	-	-	MG989005 MG988706	[16]; Percy, pers. comm.	-
Aphalarinae	*Craspedolepta anomola* (Crawford, 1914)	Canada	-	-	-	-	-	-	-	-	-	MG989006 MG988707	[16]; Percy, pers. comm.	-
Aphalarinae	*Craspedolepta bulgarica* Klimaszewski, 1961	Bulgaria, Western Stara planina Mts., Buhovo	*Achillea* sp.	42.7703	23.5663	743	10 June 2023	M	BFUS-I-IG026794	PSYBG025-23	BOLD:AFM9464	**PQ109753**	This study	Figure 1n
Aphalarinae	* *Craspedolepta conspersa* (Löw, 1888)	Czech Republic, South Moravia, Sedlec	*Artemisia vulgaris* L.	48.7746	16.6998	178	25 June 2023	F	BFUS-I-IG026829	PSYBG026-23	BOLD:AFM9463	**PQ109754**	This study	-
Aphalarinae	*Craspedolepta innoxia* (Foerster, 1848)	Bulgaria, Western Stara planina Mts., Buhovo	-	42.7703	23.5663	743	10 June 2023	F	BFUS-I-IG026795	PSYBG027-23	BOLD:AFM9464	**PQ109756**	This study	-
Aphalarinae	* *Craspedolepta malachitica* (Dahlbom, 1851)	Bulgaria, Western Stara Planina Mts., Negovan, lake	*Artemisia absinthium* L.	42.7660	23.4007	513	14 July 2022	F	BFUS-I-IG026799	PSYBG030-23	BOLD:AFL5094	**PQ109759**	This study	-
		Bulgaria, Western Stara Planina Mts., Negovan, lake		42.7660	23.4007	513	14 July 2022	F	BFUS-I-IG026992	PSYBG106-24	-	**PQ100062**	This study	-
Aphalarinae	*Craspedolepta nebulosa* (Zetterstedt, 1828)	Bulgaria, Rila-Rhodopi Massif, Rila Mts., Maljovitsa	*Chamaenerion angustifolium* (L.) Scop.	42.2083	23.3889	1750	23 July 2021	F	BFUS-I-IG026801	PSYBG032-23	BOLD:AFM9466	**PQ109760**	This study	-
		Bulgaria, Rila Mts, Maljovitsa hut		42.1880	23.3736	2010	15 June 2019	M	BFUS-I-IG026998	PSYBG107-24	-	**PQ100063**	This study	-
Aphalarinae	*Craspedolepta nervosa* (Foerster, 1848)	Bulgaria, Western Stara planina Mts., Buhovo	*Achillea* sp.	42.7703	23.5663	743	10 June 2023	F	BFUS-I-IG026796	PSYBG036-23	BOLD:ACY1733	**PQ109765**	This study	-
		Bulgaria, Western Stara planina Mts., Buhovo		42.7703	23.5663	743	10 June 2023	F	BFUS-I-IG026987	PSYBG108-24	-	**PQ100064**	This study	-
Aphalarinae	*Craspedolepta omissa* Wagner, 1944	Bulgaria, Rila-Rhodopi Massif, Rila Mts., Kartala district	*Artemisia vulgaris* L.	42.04232	23.36638	1464	2 August 2020	M	BFUS-I-IG026806	PSYBG038-23	BOLD:AFM9465	**PQ109768**	This study	-
Aphalarinae	*Craspedolepta pontica* Dobreanu & Manolache, 1962	Bulgaria, Maleshevska Planina Mts., Stara Kresna, tourist shelter	*Achillea clypeolata* Sibth. & Sm.	41.7691	23.1758	560	7 May 2022	M	BFUS-I-IG026808	PSYBG040-23	BOLD:AFM1102	**PQ109770**	This study	-
		Bulgaria, Maleshevska Planina Mts., road to Stara Kresna		41.7656	23.1659	350	30 April 2023	F	BFUS-I-IG026985	PSYBG109-24	-	**PQ100065**	This study	Figure 1r
Aphalarinae	*Craspedolepta subpunctata* (Foerster, 1848)	Bulgaria, Rila Mts, Alen mak hotel	*Chamaenerion angustifolium* (L.) Scop.	42.2121	23.3870	1712	14 June 2019	M	BFUS-I-IG026811	PSYBG043-23	BOLD:AAV0240	**PQ109771**	This study	-
		Bulgaria, Rila Mts., Alen mak hotel		42.2121	23.3870	1712	14 June 2019	M	BFUS-I-IG026989	PSYBG114-24	-	**PQ100066**	This study	-
Aphalarinae	*Lanthanaphalara mira* Tuthill, 1959	Peru	-	-	-	-	-	-	-	-	-	NC038111	[16]; Percy, pers. comm.	-
Aphalarinae	*Limataphalara lautereri* Burckhardt & Queiroz, 2013	Brazil	-	-	-	-	-	-	-	-	-	MG988785 MG989094	[16]; Percy, pers. comm.	-
Aphalarinae	*Neaphalara fortunae* Brown & Hodkinson, 1988	Costa Rica	-	-	-	-	-	-	-	-	-	MG988801 MG989115	[16]; Percy, pers. comm.	-
Aphalarinae	*Rhodochlanis bicolor* (Scott, 1880)	Bulgaria, Black Sea coast, Pomorie, salt lake	*Salicornia europaea* (Moss) Lambinon & Vanderp.	42.5998	27.6261	16	23 July 2022	M	BFUS-I-IG026956	PSYBG099-24	BOLD:AFT3392	**PQ109781** **PQ100069**	This study	Figure 1h
Rhinocolinae	*Agonoscena atlantica* Bastin, Burckhardt & Ouvrard, 2023	Canary Islands	-	-	-	-	-	-	-	-	-	OR027209 OR067180	[54]	-
Rhinocolinae	* *Agonoscena cisti* (Puton, 1882)	Albania, Memoraq	*Pistacia lentiscus* L.	39.8522	20.1470	190	10 June 2022	F	BFUS-I-IG027025	PSYBG098-24	BOLD:AEB2674	**PQ109723** **PQ100051**	This study	-
Rhinocolinae	*Agonoscena pistaciae* Burckhardt & Lauterer, 1989	Bulgaria, Eastern Rila-Rhodopi Massif, Gaberovo, Gjurgena	*Pistacia terebinthus* L.	41.6206	25.8851	280	27 August 2022	F	BFUS-I-IG026831	PSYBG067-23	BOLD:AFM5846	**PQ109726**	This study	-
Rhinocolinae	*Agonoscena sinuata* Bastin, Burckhardt & Ouvrard 2023	Canary Islands	-	-	-	-	-	-	-	-	-	OR027214 OR067162	[54,55]	-
Rhinocolinae	* *Agonoscena targionii* (Lichtenstein, 1874)	Bulgaria, Maleshevska Planina Mts., Kresna, Peyo Yavorov station	*Pistacia terebinthus* L.	41.7482	23.1615	217	13 August 2022	M	BFUS-I-IG026813	PSYBG111-24	BOLD:AFL7612	**PQ109729** **PQ100052**	This study	Figure 1a
Rhinocolinae	*Apsylla cistellata* (Buckton, 1896)	Madagascar	-	-	-	-	-	-	-	-	-	MG988642 MG988918	[16]; Percy, pers. comm.	-
Rhinocolinae	*Lisronia echidna* Loginova, 1976	Canary Islands	-	-	-	-	-	-	-	-	-	OR864739 OR067189	[55]	
Rhinocolinae	*Megagonoscena gallicola* Burckhardt & Lauterer, 1989	Bulgaria, Maleshevska Planina Mts., Stara Kresna	*Pistacia terebinthus* L.	41.7650	23.1662	360	7 May 2022	M	BFUS-I-IG026820	PSYBG051-23	BOLD:AFN1666	**PQ109777**	This study	Figure 1c
		Bulgaria, Maleshevska Planina Mts., Stara Kresna		41.7650	23.1662	360	7 May 2022	F	BFUS-I-IG027016	PSYBG112-24	-	**PQ100067**	This study	Figure 1c
Rhinocolinae	*Rhinocola aceris* (Linnaeus, 1758)	Bulgaria, Western Stara Planina Mts., Churek	*Acer* sp.	42.7805	23.7137	817	21 May 2022	M	BFUS-I-IG026822	PSYBG055-23	BOLD:ACK6660	**PQ109778**	This study	-
		Bulgaria, Transitional region, Lozenska Mts., Lozen		42.5944	23.5081	768	27 July 2022	M	BFUS-I-IG026979	PSYBG113-24	-	**PQ100068**	This study	Figure 1i
Phacopteroninae	*Pseudophacopteron* sp.	Australia	-	-	-	-	-	-	-	-	-	MG989234	[16]; Percy, pers. comm.	-
Spondyliaspidinae	*Anoeconeossa unicornuta* Taylor, 1987	Australia	-	-	-	-	-	-	-	-	-	NC_038108	[16]; Percy, pers. comm.	-
Spondyliaspidinae	*Australopsylla* sp.	Australia	-	-	-	-	-	-	-	-	-	MG988646 MG988933	[16]; Percy, pers. comm.	-
Spondyliaspidinae	*Blastopsylla occidentalis* Taylor, 1985	Australia	-	-	-	-	-	-	-	-	-	NC_038147	[16]; Percy, pers. comm.	-
Spondyliaspidinae	*Boreioglycaspis melaleucae* Moore, 1964	Australia	-	-	-	-	-	-	-	-	-	MG988659 MG988952	[16]; Percy, pers. comm.	-
Spondyliaspidinae	*Cardiaspina retator* Taylor, 1962	Australia	-	-	-	-	-	-	-	-	-	MG988694 MG988991	[16]; Percy, pers. comm.	-
Spondyliaspidinae	*Creiis* sp.	Australia	-	-	-	-	-	-	-	-	-	MG988715 MG989015	[16]; Percy, pers. comm.	-
Spondyliaspidinae	*Ctenarytaina eucalypti* (Maskell, 1890)	Canary Islands	-	-	-	-	-	-	-	-	-	OR068450 OR067182	[55]	-
Spondyliaspidinae	*Glycaspis brimblecombei* Moore, 1964	Canary Islands	-	-	-	-	-	-	-	-	-	OR068451 OR067183	[55]	-
Spondyliaspidinae	*Lasiopsylla rotundipennis* Froggatt, 1900	Australia	-	-	-	-	-	-	-	-	-	MG988781 MG989090	[16]; Percy, pers. comm.	-
Spondyliaspidinae	*Platyobria* sp.	Australia	-	-	-	-	-	-	-	-	-	MG988812 MG989131	[16]; Percy, pers. comm.	-
Liviidae, Euphyllurinae	*Psyllospsis fraxini* (Linnaeus, 1758)	United Kingdom	-	-	-	-	-	-	-	-	-	MG988820 MG989139	[16]; Percy, pers. comm.	-
Psyllidae, Psyllinae	*Cacopsylla melanoneura* (Foerster, 1848)	Italy	-	-	-	-	-	-	-	-	-	OQ304120	[56]	-
		Czech Republic	-	-	-	-	-	-	-	-	-	OR346833	[57]	-

**Table 2:** PCR primers and conditions.

Gene	Primer Set	Primer Sequence′ (5′–3′)	Amplicon Size (bp)	PCR Conditions	Primer References
*COI*	LCOP-F	AGAACWAAYCATAAAAYWATTGG	654	95 °C for 3 min; 35 cycles of 95 °C for 30 s, 50 °C 30 s and 72 °C for 1 min; 72 °C for 10 min	[54]
	HCO2198R	TAAACTTCAGGGTGACCAAAAAATCA			
	C_LepFolF	ATTCAACCAATCATAAAGATATTGG	658	94 °C for 1 min, 5 cycles of 94 °C for 30 s, 45–50 °C for 40 s, 72 °C for 1 min, 30–35 cycles of 94 °C for 30 s, 51–54 °C for 40 s and 72 °C for 1 min, 72 °C for 10 min	[58]
	C_LepFolR	TAAACTTCTGGATGTCCAAAAAATCA			
	LCO1490F	GGTCAACAAATCATAAAGATATTGG	654	95 °C for 3 min; 35 cycles of 95 °C for 30 s, 51 °C 30 s and 72 °C for 1 min; 72 °C for 10 min	[58]
	HCO2198R	TAAACTTCAGGGTGACCAAAAAATCA			
	VpmCOIF2	TACCTYTGAATTTGCAATTC	646	95 °C for 3 min; 35 cycles of 95 °C for 30 s, 46 °C 30 s and 72 °C for 1 min; 72 °C for 10 min	[59]
	VpmCOIR4	AATAARTGTTGGTATAARATAGG			
*Cytb*	Cytbf	TGAGGNCAAATATCHTTYTGA	393	95 °C for 3 min; 35 cycles of 95 °C for 30 s, 53 °C 30 s and 72 °C for 1 min; 72 °C for 10 min	[16,49]
	Cytbr	GCAAATARRAARTATCATTCDG			
	CytbnewF2	TGATTATGRGGAGGDTTYGC	330	95 °C for 3 min; 35 cycles of 95 °C for 30 s, 53 °C 30 s and 72 °C for 1 min; 72 °C for 10 min	This study
	CytbnewR	GTTGAATATGDATDGGDGTWAC			
	CytbnewF1	TATGAGGAGGDTTYGCWGTTG	248	95 °C for 3 min; 35 cycles of 95 °C for 30 s, 53 °C 30 s and 72 °C for 1 min; 72 °C for 10 min	This study
	CytbnewR	GTTGAATATGDATDGGDGTWAC			

### 2.3. Species Delimitation Based on Molecular Data

In order to verify the identification of the aphalarids, several species delimitation methods were applied to the molecular data. The Barcode Index Number (BIN) system was used to assign unique identifiers (BINs), corresponding to operational taxonomic units (OTUs), after the *COI* sequences were subjected to Refined Single Linkage (RESL) analysis in the BOLD system [60]. In addition, to evaluate the genetic distances and compare our results with those of other studies that did not use the BIN system, the Kimura 2-parameter (K2P) model was calculated for each *COI* and *Cytb* using MEGA v.11 [51]. The threshold of 3% K2P distance was used to delimit OTUs as an alternative to RESL analysis for *Cytb* data. Subsequently, Assemble Species by Automatic Partitioning (ASAP) and Automatic Barcode Gap Discovery (ABGD) methods were performed using K2P distances for each *COI* and *Cytb* [61,62]. Finally, ultrametric trees were generated using the K2P model in BEAST v. 10.5.0 [63] with 10 million replicates, followed by a 10% burn-in via TreeAnnotator. The resulting trees were saved in NEXUS format using FigTree v. 1.4.4 “http://tree.bio.ed.ac.uk/software/figtree (accessed on 8 January 2024)”, and the trees were tested with the Bayesian implementation of the Poisson Tree Processes (bPTP) [64] (implemented in the web server http://species.h-its.org/ptp/, accessed on 26 August 2024) and the multi-rate Poisson Tree Processes (mPTP) [65] (implemented in the web server http://mptp.h-its.org/#/tree, accessed on 26 August 2024).

### 2.4. Phylogenetic Analyses

In addition to the 25 aphalarid species sequenced in the current study, a further 21 species from the subfamilies Aphalarinae (6), Phacopteroninae (1), Rhinocolinae (4) and Spondyliaspidinae (10) were included in the phylogenetic analyses. *Psyllopsis fraxini* (Liviidae) and *Cacopsylla melanoneura* (Psyllidae), which are representatives of two psyllid families distantly related to Aphalaridae, were selected as outgroups.

All sequences not from this study were obtained from GenBank (Table 1). *COI* and *Cytb* sequences were extracted from the whole mitochondrial genome for the following species: *Anoeconeossa unicornuta*, *Blastopsylla occidentalis*, *Lanthanaphalara mira*, and *Pseudophacopteron* sp., while all other representatives were selected based on the availability of their *COI* and *Cytb* sequences in GenBank. Most of the sequence data are from Percy et al. [16], Bastin et al. [54,55], and Corretto et al. [56]. We consider their identifications to be reliable.

Prior to the phylogenetic analyses, the best partitioning scheme was determined using PartitionFinder2 [66], and the concatenated dataset was divided into four partitions according to gene regions and codon position. Setting of codon position for protein-coding gene fragments was performed with MESQUITE v.3.61 [67].

Maximum likelihood (ML) analysis was performed with IQ-TREE v.1.6.12. [68]. Nodal support was obtained through a standard non-parametric bootstrap with 1000 replicates. A suitable substitution model for each partition was calculated by IQ-TREE. Bayesian inference (BI) analysis was run using MrBayes v.3.2.7a [69] on the CIPRES platform [70]. The best substitution model for each gene was estimated using jModelTest v.2.1.10 [71] and a comparison of scores from Akaike information criterion (AIC) and Bayesian information criterion (BIC) (Table S2). For the BI analysis, two independent runs were performed, each with four Metropolis-coupled Markov chains for 15 million generations, sampling every 1000th generation. The priors for tree topology and speciation rates were set to uniform, the gamma distribution category number was set to 4, and the probability distribution for branch lengths was set to exponential. A Dirichlet prior was used to cover the substitution rates of the evolutionary models and the stationary nucleotide frequencies. The priors for all other parameters were left at the default values. Nodal support was assessed using posterior probabilities after discarding 30% of the samples as burn-in. The convergence of the MCMC chains was assessed with the average standard deviation of split frequencies (ASDSF). The ML and BI trees were visualised using iTOL v.5 [72] and FigTree v.1.4.4 “http://tree.bio.ed.ac.uk/software/figtree (accessed on 20 January 2024)”. Nodal support for ML analysis was assessed by the frequency of clade occurrence across the resampled datasets and is expressed as bootstrap values in %, while nodal support for BI analysis was expressed as posterior probability (PP), which indicates the probability that a given clade is correct based on the observed data and the specified model and ranges from 0 to 1. We considered significantly high nodal support for bootstrap values (BS) > 90% and for posterior probabilities (PP) > 0.95, and moderately good support for BS > 70–90% and for PP > 0.90–0.94 [73].

## 3. Results

### 3.1. Molecular Identification of the Bulgarian Aphalarids

We sequenced 25 species (1–6 specimens per species) of Aphalaridae, belonging to the two subfamilies Aphalarinae and Rhinocolinae with four and three genera, respectively, which were identified morphologically. The DNA of most analysed species was successfully amplified using the applied protocols. The *COI* gene fragment was amplified in 25 species, while *Cytb* was only successfully amplified in 18 species.

DNA barcodes were generated for the first time for the following 15 species: *Aphalara affinis*, *Aph. freji*, *Aph. nigrimaculosa*, *Colposcenia aliena*, *Co. bidentata*, *Co. osmanica*, *Co. traciana*, *Craspedolepta bulgarica*, *Cr. conspersa*, *Cr. innoxia*, *Cr. malachitica*, *Cr. omissa*, *Cr. pontica*, *Megagonoscena gallicola*, and *Rhodochlanis bicolor*.

All sequences of *COI* generated in the present study are available in BOLD (generic address), and the sequences of *COI* and *Cytb* of the analysed species are available in GenBank “https://www.ncbi.nlm.nih.gov/genbank/ (accessed on 30 July 2024)” (see also Data Availability Statement).

The Barcode Index Number (BIN) implemented in the BOLD system showed that our 61 *COI* sequences from 25 species are grouped in 22 BINs. Unique new BINs were generated for 15 species, and eight species were assigned to eight non-unique, pre-existing BINs. The majority of *COI* sequences generated were over 500 bp in length. Within Aphalarinae, the newly generated BIN BOLD:AFM9464 contained the 658 bp sequences of *Aphalara maculipennis* and *Aph. nigrimaculosa*, respectively. In addition, one of the pre-existing BINs (BOLD:ACY0265) contained the following three species: *Aph. avicularis*, *Aph. freji*, and *Aph. polygoni*. *Aphalara polygoni* from the current study was assigned to a separate, newly generated BIN (BOLD:AFL6229). In *Colposcenia*, *Co. aliena* (BOLD:AFN1581), *Co. bidentata* (BOLD:AFN1583), and *Co. traciana* (BOLD:AFL7588) were assigned new unique BINs, while *C. osmanica* was assigned to a pre-existing BIN (BOLD:AFN1582). In fact, as a result of our study we can identify the species assigned to the latter as *Cr. osmanica*. In *Craspedolepta*, all species received separate BINs, with the sole exception of *Cr. bulgarica* and *Cr. innoxia*, which were grouped together in the same BIN (BOLD:AFM9464). In *Agonoscena* (Rhinocolinae), *A. pistaciae* received two BINs. One of the sequences was assigned to a newly generated BIN (BOLD:AFL7611), while the other sequences were assigned to an already existing BIN (BOLD:AFM5846).

In addition to the BIN (RESL) analysis, we performed analyses of K2P genetic distance for both *COI* and *Cytb* gene fragments separately (Table S1). In Aphalarinae, rather high genetic distances for *COI* were observed between *Aphalara affinis* and *Aph. maculipennis* (0.1644), while a very low distance-based threshold was observed between *Aph. avicularis* and *Aph. freji* (0.0026). In *Colposcenia*, the interspecific distance for *COI* ranged from 0.1622 to 0.2365. Within the *Craspedolepta* species analysed, there were comparatively high genetic distances between *Cr. conspersa* and *Cr. nervosa* (0.1823), in contrast to the threshold between *Cr. bulgarica* and *Cr. innoxia* (0.0015), with the lowest genetic distance observed in our study. Within *Agonoscena* (Rhinocolinae), the genetic distance for *COI* was highest between *A. pistaciae* and *A. targionii* (0.2430), while the lowest genetic distance was found between *A. cisti* and *A. pistaciae* (0.1736).

**Figure 2:**
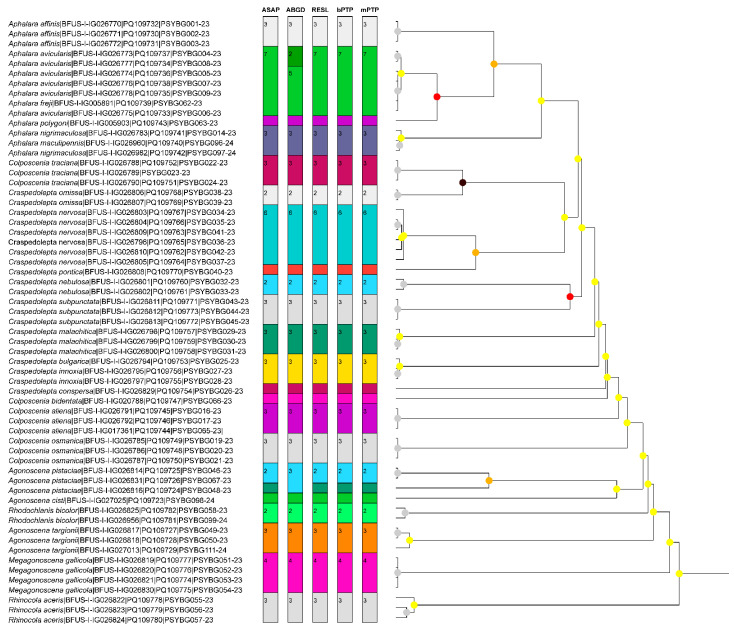
Results of species delimitation methods based on the cytochrome c oxidase subunit I (*COI*) gene. Each vertical colour bar represents different delimitation schemes obtained with ASAP, ABGD, RESL, bPTP, and mPTP methods, with the corresponding number of specimens. The tree is based on ASAP analysis, with nodes colour coded depending on their *p*-value (black: *p* < 0.001, red: *p* < 0.05, orange: *p* < 0.1, yellow: *p* > 0.1, grey: not applicable).

In addition, the species boundaries based on the *COI* marker were tested with ASAP, ABGD, bPTP, and mPTP analyses (Figure 2). All analyses consistently combined *Aphalara avicularis* with *Aph. freji*, *Aph. maculipennis* with *Aph. nigrimaculosa*, and *Craspedolepta bulgarica* with *Cr. innoxia*, indicating that the *COI* gene is unsuitable for the delimitation of these species. Similar to the results of the BIN (RESL) analysis, almost all delimitation analyses separated *A. pistaciae* into two distinct species groups, highlighting the need for a more thorough molecular and morphological investigation of *Agonoscena* spp. in future studies. The only analysis that clustered *A. pistaciae* sequences together was ABGD. However, the same analysis suggested the separation of *Aph. avicularis* into two species. In total, only 17 OTUs could then be delimited using the *COI* marker.

**Figure 3:**
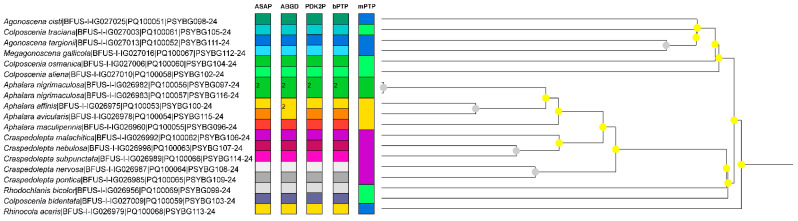
Results of species delimitation methods based on the cytochrome b (*Cytb*) gene. Each vertical colour bar represents different delimitation schemes obtained with ASAP, ABGD, bPTP and mPTP methods, with the corresponding number of specimens. Grouping based on a threshold of 3% using the Kimura 2-parameter pairwise distance (PDK2P) is presented as an alternative to RESL analysis of *COI* data. The tree is based on ASAP analysis, with nodes colour coded depending on their *p*-values (yellow: *p* > 0.1, grey: not applicable).

With respect to *Cytb*, the interspecific divergence within the genera analysed was usually somewhat higher than that observed for *COI*. The maximum genetic distance within *Aphalara* was 0.1681 between *Aph. avicularis* and *Aph. nigrimaculosa*, while the distance between *Aph. affinis* and *Aph. avicularis* was the smallest (0.0771) of the species pairs that could be compared. In *Colposcenia* and *Craspedolepta*, the interspecific divergence ranged from 0.2571 to 0.3243 and from 0.1108 to 0.2803, respectively (however, *Cytb* data were available only for some *Craspedolepta* spp.). In *Agonoscena*, the interspecific distance between *Agonoscena cisti* and *A. pistaciae* was 0.2598. In most cases, the *Cytb* gene fragments gave comparable or better results for the separation of Aphalaridae species than the *COI* gene fragments. Using the 3% genetic distance threshold (as an alternative to RESL analysis for *COI*), ASAP, and bPTP, all 18 species analysed were successfully delimited. However, ABGD produced slightly different results and merged *Aphalara affinis* and *Aph. avicularis* into a single cluster, while mPTP was less effective and failed to delimit the taxa to species level, with the exception of *Aph. nigrimaculosa*, and instead grouped them into five clusters at the subfamily or genus level (Figure 3).

### 3.2. Phylogeny of Aphalaridae

**Figure 4:**
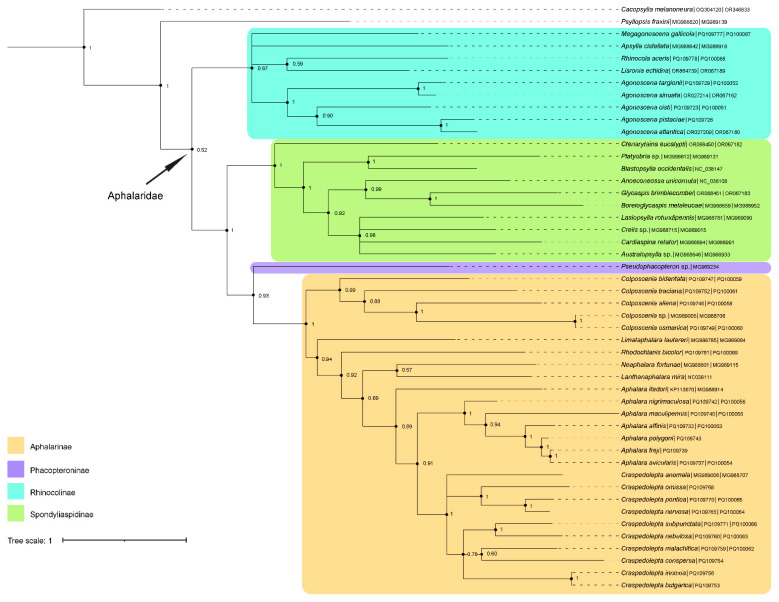
Phylogenetic tree based on Bayesian inference (BI) analysis of concatenated *COI* and *Cytb* gene fragments of representatives of the family Aphalaridae. Nodal support is given as posterior probability values.

In addition to the 25 aphalarid species sequenced in the current study, 21 species from the subfamilies Aphalarinae, Phacopteroninae, Rhinocolinae, and Spondyliaspidinae were included in the phylogenetic analyses. The final sequence alignment of 46 species of Aphalaridae and two outgroups contained 1043 characters. As both Bayesian inference (BI) and maximum likelihood (ML) analysis yielded very similar results, only the phylogenetic tree from the BI is shown in Figure 4 in the main text, while the ML tree is included as a Supplementary File (Figure S1).

In both analyses, support for monophyly of Aphalaridae was weak or virtually absent (0.52 PP, 24% BS). The subfamily Aphalarinae was strongly supported in both BI and ML analyses (1.00 PP, 95% BS). *Aphalara* was recovered as paraphyletic (0.89 PP, 66% BS), with *Aph. itadori* forming the sister taxon to the remaining *Aphalara* and *Craspedolepta*. The internal relationships of *Aphalara* were well supported in BI (0.94–1.00 PP) and ML analyses (92–99% BS), with the exception of *Aph. itadori* (66% BS) and *Aph. maculipennis* (74% BS). *Craspedolepta* was a strongly supported clade in both BI and ML analyses (1.00 PP, 96% BS). Four clades were strongly supported within the genus: *Cr. omissa* + (*Cr. nervosa* + *Cr. pontica*) (1.00 PP, 97% BS); *Cr. nervosa* + *Cr. pontica* (1.00 PP, 98% BS); *Cr. nebulosa* + *Cr. subpunctata* (1.00 PP, 96% BS); and *Cr. bulgarica + Cr. innoxia* (1.00 PP, 100% BS).

The support for monophyly of Rhinocolinae was weak in the ML analysis (72% BS) but strong in the BI (0.97 PP). *Agonoscena* was strongly supported in both analyses (1.00 PP, 90% BS), while support for the other genera was moderate to weak.

A sister group relationship between Phacopteroninae, represented only by *Pseudophacopteron* sp., and Aphalarinae was moderately supported (0.93 PP, 85% BS). The phylogenies of the two analyses also shared the same topology in terms of the strongly supported Spondyliaspidinae (1.00 PP, 97% BS) with a strongly supported clade *Blastopsylla occidentalis* Taylor, 1985 + *Platyobria* sp. (1.00 PP, 96% BS). The other nodes of the clade Spondyliaspidinae were weakly to moderately supported in the ML analysis. In the BI, *Anoeconeossa unicornuta* was strongly supported (0.99 PP) as a sister species to the clade *Boreioglycaspis melaleucae* + *Glycaspis brimblecombei* (1.00 PP). *Lasiopsylla rotundipennis*, *Australopsylla* sp., and *Creiis* sp. together constituted, with strong support (0.98 PP), the sister clade to *Cardiaspina retator*.

### 3.3. Faunistic Data

The following five species represent new records for Bulgaria: *Agonoscena targionii*, *Aphalara affinis*, *Colposcenia aliena*, *Co. bidentata* and *Craspedolepta malachitica*. *Craspedolepta conspersa* is reported for the first time from the Czech Republic, while *Agonoscena cisti* is reported for the first time from Albania (Table 1).

## 4. Discussion

### 4.1. Molecular Identification of Species

The highest species diversity among the psyllids is probably found in tropical and southern temperate regions [14], but there are also some characteristic northern temperate genera, such as *Psylla* Geoffroy and *Spanioneura* Foerster (Psyllidae) [14] or the species-rich *Aphalara* and *Craspedolepta* [32] (Aphalaridae). The latter family comprises about 60 described species in Europe, about half of which also occur in Bulgaria [15]. *Aphalara* and *Craspedolepta* are particularly diverse in Bulgaria, with seven and eleven species, respectively. The taxonomic knowledge of the two genera, based on morphology, is good for Europe [37], fair for the rest of the Palaearctic (e.g., [32,40,41]), but poor for the Nearctic [74]. Some species groups are morphologically homogeneous, which makes species identification difficult for non-experts. Here, we show that most of the 25 studied aphalarid species from seven genera, including *Aphalara* and *Craspedolepta*, differ significantly in molecular barcodes (*COI* and *Cytb*), which can be used to identify species that are difficult to separate morphologically. The DNA barcodes for 15 species are published here for the first time.

In other similar studies on psyllids, a distance-based threshold of 3% for mitochondrial markers was suggested as appropriate for distinguishing species of psyllids in New Zealand [2] or *Cacopsylla* species developing on pear in the Palaearctic [8]. We found a good correlation between molecular, morphological, and biological evidence for species delimitation, with a relatively high genetic divergence for most species supporting the 3% threshold. We suggest here that the *COI* and *Cytb* barcoding genes are useful for the molecular identification of most aphalarid species. However, the *COI* gene fragment alone was not sufficient to clearly separate six morphologically supported species of *Aphalara* and *Craspedolepta* in our study. Some other studies, e.g., on grasshoppers and fungus gnats, comparing morphological and molecular species identification [75,76] also showed contradictory results, with morphologically distinct taxa being indistinguishable in a single gene fragment.

The observed small interspecific difference in the *COI* between *Aphalara avicularis* and *Aph. freji* (0.0026), which could be due to genuine genetic proximity or limitations in the resolution of the *COI* gene, is also reflected in the morphological similarities between these two species [32,37]. Within *Aphalara*, there are several morphologically homogeneous species groups whose species differ in the distribution of surface spinules on the forewings and often subtle details of the terminalia [32]. These differences are paralleled by the host ranges. *Aphalara avicularis* and *Aph. freji* develop on the *Polygonum aviculare* aggregate and *Persicaria* spp., respectively, and differ from each other by the shape of the distal segment of the aedeagus and, to a certain extent, in the spacing of the surface spinules on the forewings. In the morphologically similar and closely related *Aph. polygoni*, which develops on *Rumex* spp., the surface spinules of the forewings are arranged in transverse rows. Two congeneric but morphologically different species, *Craspedolepta bulgarica* and *Cr. innoxia*, also showed only a slight genetic divergence. In morphology and host plant association [77], *Cr. bulgarica* is more similar to *Cr. nervosa* and *Cr. pontica*, which all develop on *Achillea* spp., than *Cr. innoxia*, which is associated with *Daucus carota* and *Seseli leucospermum* [78]. On the other hand, the interspecific genetic distances based on the *COI* for *Agonoscena* were significantly higher in our study than those found by Lashkari, et al. [47] for a different set of species in Iran (*A. bimaculata*, *A. pegani*, and *A. pistaciae*). Further studies on the genus are needed to clarify whether these discrepancies are due to differences in methodology, sample selection, or actual genetic divergence between species. All species delimitation methods based on *COI* yielded similar results and recognised 17 of 25 aphalarid species. However, the ABGD analysis yielded some contradictory results, suggesting cryptic lineages within *Aphalara avicularis*. This interpretation seems unlikely, as all other species delimitation methods and morphological observations consistently supported the conclusion that the *Aph. avicularis* specimens belong to a single species. On the other hand, the ABGD was the only method that recognised *Agonoscena pistaciae* specimens as a single species, which is consistent with morphological evidence.

In contrast to the *COI* delimitation results, where a maximum of 68% of the species were recognised, the *Cytb* gene allowed the correct assignment of all 100% of the species analysed using the 3% genetic distance threshold, ASAP, and bPTP, suggesting that this gene is an efficient marker for the recognition of aphalarid species, which was already suggested for *Agonoscena* by Lashkari et al. [47]. As we did not sequence *Cytb* for all species in this study, the comparison of the two genes remains preliminary.

### 4.2. Phylogenetic Relationships within Aphalaridae

The phylogenetic results of our study on Aphalaridae are similar to other phylogenetic analyses based on genomic or multilocus DNA sequence data [16,17]. The phylogenetic reconstruction inferred by the BI and ML analyses resulted in a similar tree topology, albeit with some differences (Figure 4 and Figure S1). In both analyses we performed, Aphalaridae were recovered as monophyletic, albeit with very low or virtually absent clade support. In their comprehensive amino-acid-based analysis, Wang et al. [18] found Aphalaridae to be monophyletic, in contrast to Percy et al. [16] and Cho et al. [17], who considered the family as paraphyletic.

In several studies [16,17,23,24,25], the monophyly of Aphalarinae and Rhinocolinae was well supported. Similarly, both subfamilies were strongly supported in our BI analysis. In contrast, the ML analysis in our study showed strong support for Aphalarinae, while Rhinocolinae was only weakly supported. Our analyses strongly supported the sister-group relationship between the Spondyliaspidinae and the moderately supported clade Aphalarinae + Phacopteroninae. Representatives of the three small subfamilies of Aphalaridae (Cecidopsyllinae, Microphyllurinae, and Togepsyllinae) were not included in our analyses, which may partly explain deviations from the previously published tree topologies [14,16,17,18].

Within Aphalarinae, our results are generally consistent with these from the previous studies [16,25,32]. There are two important differences in our analyses. *Aphalara* was recovered as paraphyletic with respect to *Craspedolepta*, as *Aph. itadori* formed a sister group to the remaining *Aphalara* + *Craspedolepta*. *Craspedolepta bulgarica* formed a strongly supported clade together with *Cr. innoxia*. Based on the morphological and host plant data [32,77], both groupings are highly unlikely and could result from the use of the limited number of exclusively mitochondrial gene markers.

Within Rhinocolinae, our results are in agreement with other studies [23,24]. *Agonoscena* was strongly supported as monophyletic, and its internal relationships are consistent with those proposed by Burckhardt and Lauterer [23] based on morphology and by Bastin et al. [79] based on molecular data.

The phylogenetic results of our study, especially the higher-level relationships, must be interpreted with caution, as only two short mitochondrial gene fragments were analysed here. For a more accurate investigation of the phylogenetic relationships within the group, additional nuclear markers should be used in future studies.

### 4.3. New Data on the Distribution of Aphalarid Species

The psyllid fauna of Bulgaria has been treated in a number of works (e.g., [23,26,27,28,29,30,31,32,33,34,80]), in which less than a hundred species were reported [15,35]. The revision of existing museum collections and more intensive faunistic research in recent years have revealed additional species [35,36,81]. Here we report five species for the first time from Bulgaria. *Aphalara affinis* is a species with a boreomontane distribution in northern Europe and Siberia as well as in the mountains of Central and Eastern Europe; it is associated with *Stellaria graminea* (Caryophyllaceae) in grasslands and forest clearings [15,37,82]. In Bulgaria, *Aph. affinis* was found in the Western Rhodopi Mountains. *Colposcenia aliena* and *Co. bidentata* are both associated with *Tamarix* spp. (Tamaricaceae), which in Bulgaria usually grow on sandy or stony substrates along rivers and the Black Sea coast. *Colposcenia aliena* is widespread in southern Europe, North Africa, the Middle East, and Central Asia [15,38,83]. *Colposcenia bidentata* is so far only known from Turkey [39,84]. *Craspedolepta malachitica* is widespread in Europe and temperate Asia on *Artemisia absinthium* and *A. maritima* (Asteraceae) in open habitats, such as disturbed dry grassland or salty sites [15,37,44]. Finally, *Agonoscena targionii* is widespread in its native range in the Mediterranean Basin and was introduced to Britain and Saint Helena; it is associated with *Pistacia* spp. (*P. lentiscus*, *P. terebinthus*, and *P. vera*; Anacardiaceae) [23,85].

The psyllid fauna of the Czech Republic is quite well known, with a rather long history of investigation and a total number of over 130 recorded species [86,87,88]. Here we report for the first time on *Craspedolepta conspersa* from the Czech Republic. The species was collected on its known host plant, *Artemisia vulgaris* (Asteraceae), in a ruderal habitat in southern Moravia, the warmest part of the country. *Craspedolepta conspersa* is known from southern parts of Central and Eastern Europe [43,44] and was recently reported for the first time from neighbouring Austria [89]. The Czech record is located at the northern limit of the species’ currently known range.

Compared to Bulgaria and the Czech Republic, Albania has only been very sparsely studied for Psylloidea. *Agonoscena cisti*, which is newly recorded here from Albania, is, similar to *A. targionii*, widely distributed in the Mediterranean region on *Pistacia lentiscus* and *P. palaestina* [23,90]. Given the presence of the species in neighbouring countries, its occurrence in Albania is not surprising.

## 5. Conclusions

Our study investigated the suitability of two barcoding genes, *COI* and *Cytb*, for the identification of species from the subfamilies Aphalarinae and Rhinocolinae (Aphalaridae). We provide the DNA barcodes for 25 species collected mainly in Bulgaria, of which 15 species were barcoded for the first time. The use of correctly identified barcodes will help to improve the dubious quality of repositories, such as GenBank and BOLD in the future. We have designed new primers for *Cytb* that can be used in future studies. In our study, the *Cytb* gene fragment showed better results by correctly delimiting 100% of the analysed species, compared to *COI* (68%), and thus could be a useful tool for efficient and accurate identification of aphalarid species. Overall, we believe that molecular data are a valuable tool, providing new insights for the diagnosis of aphalarid species, especially when combined with reliable morphological characters. We found good agreement between the resulting species definitions based on molecular, morphological and ecological (e.g., host plant) data. However, the low genetic divergence between some aphalarid species found in our study also demonstrates the importance of using an integrative taxonomic approach [2,48,91], including the study of psyllid morphology, ecological data and multilocus molecular data, for accurate species identification.

## Data Availability

All DNA sequences can be accessed in BOLD under the project name “PSYBG” (Process ID: PSYBG001-23–PSYBG067-23, PSYBG096-24–PSYBG116-24), available also in GenBank (*COI* accession numbers: PQ109723–PQ109782, *Cytb* accession numbers: PQ100051–PQ100069).

## Supplementary Materials

The following supporting information can be downloaded at: https://www.mdpi.com/article/10.3390/insects15090683/s1, Figure S1: Phylogenetic tree based on the maximum likelihood analysis of concatenated *COI* and *Cytb* gene fragments of representatives of the family Aphalaridae. Nodal support is assessed by bootstrap values; Table S1: Interspecific K2P distances (*COI* and *Cytb* gene fragments) of the analysed species of Aphalaridae; Table S2: Gene partitions with estimated nucleotide substitution models used in Bayesian inference (BI) analysis based on a comparison of scores from Akaike Information Criterion (AIC) and Bayesian Information Criterion (BIC) in JModeltest.
