# Supplementary material for: Unravelling the Molecular Identity of Bulgarian Jumping Plant Lice of the Family Aphalaridae (Hemiptera: Psylloidea)
Source: Insects. 2024 Sep 10;15(9):683. doi: 10.3390/insects15090683 (PMC11431860; doi:10.3390/insects15090683)
Supplement: Supplementary file 1 [file insects-15-00683-s001.zip › Pramatarova_etal_Table_S1_K2P.pdf]

Supplementary Table S1. Interspecific K2P distances (COI and Cytb gene fragments) of the analysed Bulgarian species of Aphalaridae.

Species	*COI*	*Cytb*	Species	*COI*	*Cytb*
Ag. cisti – Ag. pistaciae	0.1736	x	*Cr. bulgarica – Cr. subpunctata*	0.1551	x
Ag. cisti – Ag. targionii	0.1950	0.2598	*Cr. conspersa – Cr. innoxia*	0.1641	x
Ag. pistaciae - Ag. targioni	0.2430	x	*Cr. conspersa – Cr. malachitica*	0.1669	x
A. affinis – A. avicularis	0.0744	0.0771	*Cr. conspersa – Cr. nebulosa*	0.1622	x
A. affinis – A. freji	0.0834	x	*Cr. conspersa – Cr. nervosa*	0.1823	x
A. affinis – A. maculipennis	0.1645	0.1493	*Cr. conspersa – Cr. omissa*	0.1754	x
A. affinis – A. nigrimaculosa	0.1248	0.1405	*Cr. conspersa – Cr. pontica*	0.1526	x
A. affinis – A. polygoni	0.0728	x	*Cr. conspersa – Cr. subpunctata*	0.1802	x
A. avicularis - A. freji	0.0026	x	*Cr. innoxia – Cr. malachitica*	0.1590	x
A. avicularis – A. maculipennis	0.1544	0.1542	*Cr. innoxia – Cr. nebulosa*	0.1514	x
A. avicularis – A. nigrimaculosa	0.1119	0.1681	*Cr. innoxia – Cr. nervosa*	0.1763	x
A. avicularis – A. polygoni	0.0298	x	*Cr. innoxia – Cr. omissa*	0.1679	x
A. freji – A. maculipennis	0.1342	x	*Cr. innoxia – Cr. pontica*	0.1662	x
A. freji – A. nigrimaculosa	0.1162	x	*Cr. innoxia – Cr. subpunctata*	0.1532	x
A. freji – A. polygoni	0.0350	x	*Cr. malachitica – Cr. nebulosa*	0.1555	0.1462
A. maculipennis – A. nigrimaculosa	0.1644	0.1654	*Cr. malachitica – Cr. nervosa*	0.1611	0.2588
A. maculipennis – A. polygoni	0.1604	x	*Cr. malachitica – Cr. omissa*	0.1739	x
C. aliena – C. bidentata	0.1622	0.2722	*Cr. malachitica – Cr. pontica*	0.1472	0.2803
C. aliena – C. osmanica	0.2156	0.2684	*Cr. malachitica – Cr. subpunctata*	0.1790	0.1618
C. aliena – C. traciana	0.1905	0.3167	*Cr. nebulosa – Cr. nervosa*	0.1594	0.2057
C. bidentata – Cr. osmanica	0.1818	0.3243	*Cr. nebulosa – C. omissa*	0.1742	x
C. bidentata – Cr. traciana	0.1864	0.2711	*Cr. nebulosa – C. pontica*	0.1418	0.2350
C. osmanica – Cr. traciana	0.2365	0.2571	*Cr. nebulosa – C. subpunctata*	0.1315	0.1108
Cr. bulgarica – Cr. conspersa	0.1661	x	*C. nervosa – C. omissa*	0.1328	x
Cr. bulgarica – Cr. innoxia	0.0015	x	*C. nervosa – C. pontica*	0.0630	0.1297
Cr. bulgarica – Cr. malachitica	0.1610	x	*C. nervosa – C. subpunctata*	0.1494	0.2053
Cr. bulgarica – Cr. nebulosa	0.1534	x	*C. omissa – C. pontica*	0.1231	x
Cr. bulgarica – Cr. nervosa	0.1783	x	*C. omissa – C. subpunctata*	0.1531	x
Cr. bulgarica – Cr. omissa	0.1699	x	*C. pontica – C. subpunctata*	0.1420	0.2228
Cr. bulgarica – Cr. pontica	0.1662	x			
