# Supplementary material for: Unravelling the Molecular Identity of Bulgarian Jumping Plant Lice of the Family Aphalaridae (Hemiptera: Psylloidea)
Source: Insects. 2024 Sep 10;15(9):683. doi: 10.3390/insects15090683 (PMC11431860; doi:10.3390/insects15090683)
Supplement: Supplementary file 1 [file insects-15-00683-s001.zip › Pramatarova_et_al_Table_S2_Gene_partitions_BI_analysis.pdf]

Supplementary Table S2. Gene partitions with estimated nucleotide substitution models used in Bayesian inference (BI) analysis based on a comparison of scores from Akaike Information Criterion (AIC) and Bayesian Information Criterion (BIC) in JModeltest.

Gene partitions	Substitution models
COI = 1-654\3, 2-654\3	GTR + I + gamma
COI = 3-654\3	GTR + gamma
cytb = 655-1043\3, 656-1043\3	GTR + gamma
cytb = 657-1043\3	GTR + gamma
