# Supplementary figures and images for: Unravelling the Molecular Identity of Bulgarian Jumping Plant Lice of the Family Aphalaridae (Hemiptera: Psylloidea)
Source: Insects. 2024 Sep 10;15(9):683. doi: 10.3390/insects15090683 (PMC11431860; doi:10.3390/insects15090683)

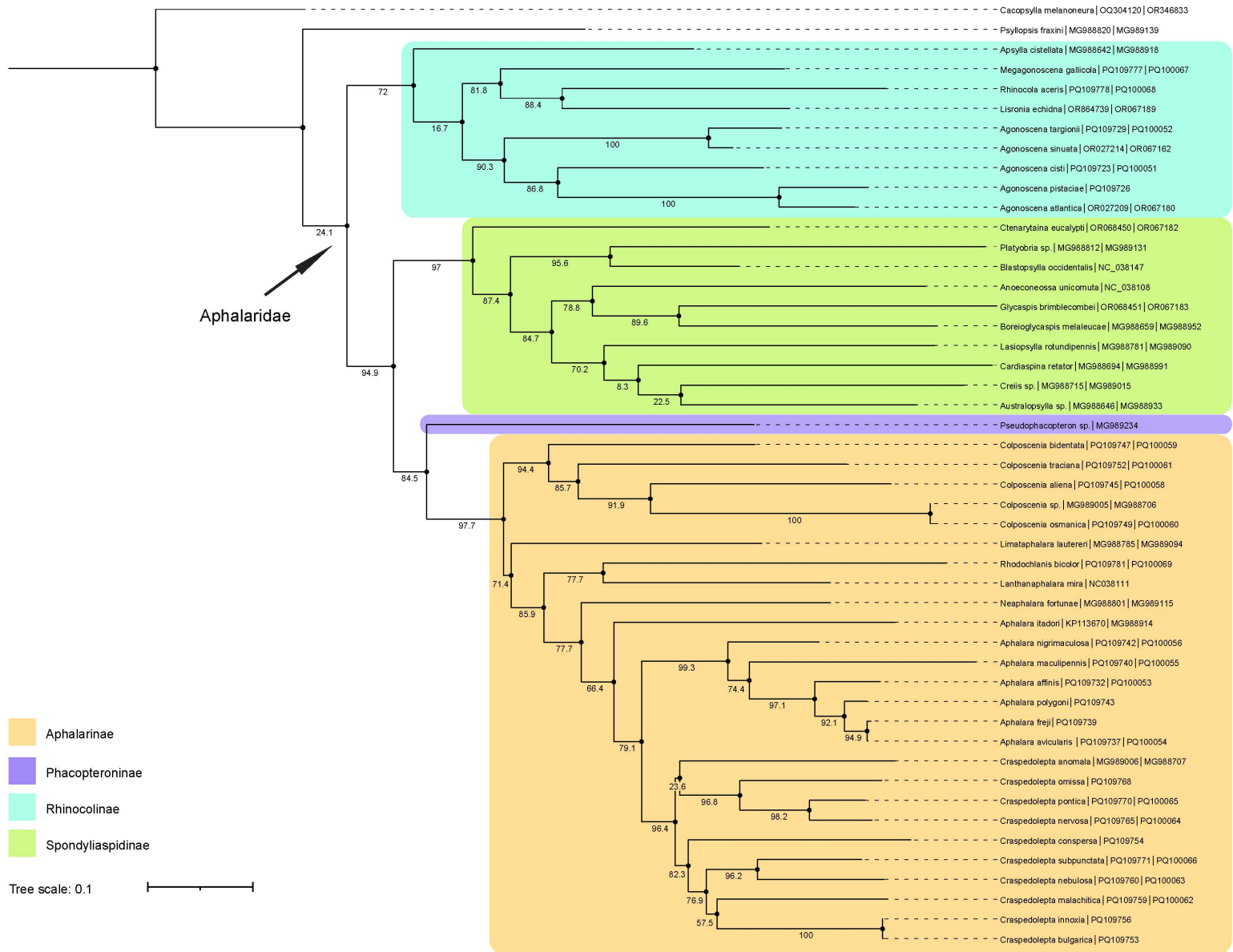

Supplement: Supplementary file 1 [file insects-15-00683-s001.zip › Fig_S1.pdf]
